# Supplementary material for: Learning physical properties of liquid crystals with deep convolutional neural networks
Source: Sci Rep. 2020 May 6;10:7664. doi: 10.1038/s41598-020-63662-9 (PMC7203147; doi:10.1038/s41598-020-63662-9)
Supplement: Supplementary file 1 — Supplementary information. [file 41598_2020_63662_MOESM1_ESM.pdf]

Supplementary Information for

Learning physical properties of liquid crystals with deep convolutional neural networks

Higor Y. D. Sigaki et al., Sci. Rep., 2020.

DEFINING THE CONVOLUTIONAL NEURAL NETWORKS OF OUR WORK WITH HIGH-LEVEL API

```
import tensorflow as tf
from tensorflow.keras import layers
from tensorflow import keras

def keras_model_for_predicting_phase(nblocks=2, nfilters=5,
                                     filter_size=2, polling_size=2,
                                     dense=[32, 16], reg_lamda=0.005):
    """
    Returns a CNN used for predicting the phase of nematic liquid crystals.

    Parameters:
    nblocks: number of convolution and max-pooling blocks.
    nfilters: number of convolution filters in each convolution block.
    filter_size: size of the convolution filters.
    polling_size: size of the pooling filters.
    dense: a list with the number of neurons in each dense layer.
    reg_lamda: L2 norm for regularization.

    Returns:
    A tf.keras model.
    """

    model = tf.keras.Sequential()
    model.add(
        layers.Conv2D(nfilters,
                      filter_size,
                      activation='relu',
                      input_shape=(100,100,1,),
                      kernel_regularizer=keras.regularizers.l2(reg_lamda)))
    model.add(layers.MaxPooling2D(pool_size=(polling_size, polling_size)))

    for i in range(nblocks - 1):
        model.add(
            layers.Conv2D(nfilters, filter_size, activation='relu',
                          kernel_regularizer=keras.regularizers.l2(reg_lamda)))
        model.add(layers.MaxPooling2D(pool_size=(polling_size, polling_size)))

    model.add(layers.Flatten())

    for ndense in dense:
        model.add(
            layers.Dense(ndense, activation='relu',
                         kernel_regularizer=keras.regularizers.l2(reg_lamda)))

    model.add(layers.Dense(1, activation='sigmoid'))

    return model
```

Code 1. Python code used for defining a function that returns a keras convolutional neural network model for predicting the phase of nematic liquid crystals.

```python
import tensorflow as tf
from tensorflow.keras import layers
from tensorflow import keras

def keras_model_for_predicting_order_parameter(nblocks=4,
                                               nfilters=5,
                                               filter_size=2,
                                               polling_size=2,
                                               dense=[32, 16],
                                               reg_lamda=0.005):
    """
    Returns a CNN used for predicting the order parameter of nematic liquid crystals.

    Parameters:
    nblocks: number of convolution and max-pooling blocks.
    nfilters: number of convolution filters in each convolution block.
    filter_size: size of the convolution filters.
    polling_size: size of the pooling filters.
    dense: a list with the number of neurons in each dense layer.
    reg_lamda: L2 norm for regularization.

    Returns:
    A tf.keras model.

    """
    model = tf.keras.Sequential()
    model.add(
        layers.Conv2D(nfilters,
                      filter_size,
                      activation='relu',
                      input_shape=(100,100,1,),
                      kernel_regularizer=keras.regularizers.l2(reg_lamda)))
    model.add(layers.MaxPooling2D(pool_size=(polling_size, polling_size)))

    for i in range(nblocks - 1):
        model.add(
            layers.Conv2D(nfilters,
                          filter_size,
                          activation='relu',
                          kernel_regularizer=keras.regularizers.l2(reg_lamda)))
        model.add(layers.MaxPooling2D(pool_size=(polling_size, polling_size)))

    model.add(layers.Flatten())

    for ndense in dense:
        model.add(
            layers.Dense(ndense,
                         activation='relu',
                         kernel_regularizer=keras.regularizers.l2(reg_lamda)))

    model.add(layers.Dense(1, activation='linear'))

    return model
```

Code 2. Python code used for defining a function that returns a keras convolutional neural network model for predicting the order parameter of nematic liquid crystals.

```python
import tensorflow as tf
from tensorflow.keras import layers
from tensorflow import keras

def keras_model_for_predicting_pitch(nblocks=4,
                                     nfilters=5,
                                     filter_size=2,
                                     polling_size=2,
                                     dense=[16],
                                     reg_lamda=0.005):
    """
    Returns a CNN used for predicting the pitch of cholesteric liquid crystals.

    Parameters:
    nblocks: number of convolution and max-pooling blocks.
    nfilters: number of convolution filters in each convolution block.
    filter_size: size of the convolution filters.
    polling_size: size of the pooling filters.
    dense: a list with the number of neurons in each dense layer.
    reg_lamda: L2 norm for regularization.

    Returns:
    A tf.keras model.

    """
    model = tf.keras.Sequential()
    model.add(
        layers.Conv2D(nfilters,
                      filter_size,
                      activation='relu',
                      input_shape=(200,200,1,),
                      kernel_regularizer=keras.regularizers.l2(reg_lamda)))
    model.add(layers.MaxPooling2D(pool_size=(polling_size, polling_size)))

    for i in range(nblocks - 1):
        model.add(
            layers.Conv2D(nfilters,
                          filter_size,
                          activation='relu',
                          kernel_regularizer=keras.regularizers.l2(reg_lamda)))
        model.add(layers.MaxPooling2D(pool_size=(polling_size, polling_size)))

    model.add(layers.Flatten())

    for ndense in dense:
        model.add(
            layers.Dense(ndense,
                         activation='relu',
                         kernel_regularizer=keras.regularizers.l2(reg_lamda)))

    model.add(layers.Dense(8, activation='softmax'))

    return model
```

Code 3. Python code used for defining a function that returns a keras convolutional neural network model for predicting the pitch of cholesteric liquid crystals.

```python
import tensorflow as tf
from tensorflow.keras import layers
from tensorflow import keras

def keras_model_for_predicting_e7_temperature(nblocks=3, nfilters=8,
                                              filter_size=4, polling_size=3,
                                              dense=[32, 16], reg_lamda=0.005):
    """
    Returns a CNN used for predicting the temperature of E7 liquid crystals.

    Parameters:
    nblocks: number of convolution and max-pooling blocks.
    nfilters: number of convolution filters in each convolution block.
    filter_size: size of the convolution filters.
    polling_size: size of the pooling filters.
    dense: a list with the number of neurons in each dense layer.
    reg_lamda: L2 norm for regularization.

    Returns:
    A tf.keras model.

    """
    model = tf.keras.Sequential()
    model.add(
        layers.Conv2D(nfilters,
                      filter_size,
                      activation='relu',
                      input_shape=(510,511,1,),
                      kernel_regularizer=keras.regularizers.l2(reg_lamda)))
    model.add(
        layers.Conv2D(nfilters,
                      filter_size - d_filter_size,
                      activation='relu',
                      kernel_regularizer=keras.regularizers.l2(reg_lamda)))
    model.add(layers.MaxPooling2D(pool_size=(polling_size, polling_size)))

    for i in range(nblocks - 1):
        model.add(
            layers.Conv2D(nfilters,
                          filter_size,
                          activation='relu',
                          kernel_regularizer=keras.regularizers.l2(reg_lamda)))
        model.add(
            layers.Conv2D(nfilters,
                          filter_size - d_filter_size,
                          activation='relu',
                          kernel_regularizer=keras.regularizers.l2(reg_lamda)))
        model.add(layers.MaxPooling2D(pool_size=(polling_size, polling_size)))

    model.add(layers.Flatten())

    for ndense in dense:
        model.add(
            layers.Dense(ndense,
                         activation='relu',
                         kernel_regularizer=keras.regularizers.l2(reg_lamda)))

    model.add(layers.Dense(1, activation='linear'))

    return model
```

Code 4. Python code used for defining a function that returns a keras convolutional neural network model for predicting the temperature of E7 liquid crystals.

```
from sklearn.metrics import r2_score
from keras import backend as K

def coeff_determination(y_true, y_pred):
    SS_res =  K.sum(K.square( y_true-y_pred ))
    SS_tot = K.sum(K.square( y_true - K.mean(y_true)))
    return (1 - SS_res/(SS_tot + K.epsilon()))

model = keras_model_for_predicting_e7_temperature()

adam = keras.optimizers.Adam(lr=0.001)

model.compile(optimizer=adam, loss='mse', metrics=[coeff_determination, 'mse'])

callback = [keras.callbacks.EarlyStopping(monitor='val_loss', patience=10)]

history = model.fit(X_train,
                    y_train,
                    epochs=500,
                    validation_data=(X_val, y_val),
                    callbacks=callback,
                    verbose=1,
                    batch_size=32)

test_predictions = model.predict(X_test)
r2_score(y_test,test_predictions)
```

Code 5. Example of Python code used training a keras convolutional neural network model for predicting the temperature of E7 liquid crystals. Here `X_train`, `X_val`, and `X_test` represent respectively the image files in the training, validation and test sets. While `y_train`, `X_val`, `y_val` represent respectively the temperature values of the training, validation and test sets.
